# The role of endoscopic ultrasound-guided fine-needle aspiration/biopsy in the diagnosis of mediastinal lesions

**DOI:** 10.3389/fsurg.2022.1065070

**Published:** 2023-01-06

**Authors:** Jingjing Zhou, Ting Cai, Dongwen Wu, Xiong Chen, Fen Wang

**Affiliations:** ^1^ Department of Gastroenterology, The Third Xiangya Hospital, Central South University, Changsha, China; ^2^ Hunan Key Laboratory of Nonresolving Inflammation and Cancer, The Third Xiangya Hospital, Central South University, Changsha, China

**Keywords:** ultrasound gastroscopy, endoscopic ultrasound-Guided fine needle aspiration, endoscopic ultrasound-guided fine needle biopsy, mediastinal lesions, diagnostic value

## Abstract

**Objective:**

Endoscopic ultrasound-guided fine-needle aspiration/biopsy (EUS-FNA/FNB) is an accurate technique for sampling the pancreas and mediastinum. The aim of this study was to determine the value of EUS-FNA/FNB in the diagnosis of mediastinal lesions.

**Methods:**

Data from 107 patients who underwent EUS-FNA/FNB for mediastinal lesions were evaluated.

**Results:**

The sensitivity, specificity, positive predictive value, and negative predictive value of EUS-FNA/FNB for mediastinal lesions were 92.00%, 100%, 100%, and 85%, respectively. The sensitivity, specificity, positive predictive value, and negative predictive value of EUS-FNA for malignant mediastinal lesions were 92.00%, 100%, 100%, and 86.00%, respectively. The sensitivity, specificity, positive predictive value, and negative predictive value of EUS-FNB for malignant mediastinal lesions were 92.00%, 100%, 100%, and 82.00%, respectively. Except for the discomfort caused by conventional gastroscopy, none of the patients had any complications, such as damage to surrounding large blood vessels or nerves.

**Conclusion:**

EUS-FNA/FNB is an effective tool for diagnosing unknown mediastinal lesions, without any obvious complications.

## Introduction

Endoscopic ultrasound-guided fine-needle aspiration/biopsy (EUS-FNA/FNB) uses a dedicated needle to accurately puncture the target under real-time monitoring by an ultrasound probe and negative pressure aspiration. The tissue is aspirated for pathological examination. There are many types of mediastinal lesions. Mediastinal malignant lesions are often seen in enlarged lymph nodes, and are lymph node metastases of various malignant tumors. Benign diseases of the mediastinum include sarcoidosis, tuberculosis, and histoplasmosis. The detection rate of CT, MRI, and EUS for mediastinal lesions can reach 14% (1). At present, clinical methods such as open thoracic biopsy under mediastinoscopy, tissue puncture under ultrasound or CT guidance, fine needle aspiration under bronchoscope guidance (TBNA), or fine needle aspiration under endoscopy (EUS-FNA/FNB) can be used to obtain a pathological diagnosis of the mediastinal space. Compared with other puncture methods, EUS-FNA/FNB has the following advantages: easy detection of small lesions, high sensitivity and specificity, able to access the posterior mediastinum/nonparatracheal lymph nodes, low risk, simple operation, low cost, and few complications (2–4). It has been reported in the literature that the accuracy of EUS-FNA in the diagnosis of solid mediastinal lesions can reach 52%–94% (5).

In this study, we retrospectively analyzed patients who underwent EUS-FNA/FNB due to mediastinal masses or mediastinal swelling. The diagnostic value and clinical significance of EUS-FNA/FNB for mediastinal space-occupying and mediastinal enlarged lymph nodes were explored.

## Materials and methods

### General information

A retrospective study of patients admitted to endoscopic ultrasound-guided fine needle aspiration/biopsy at the Third Xiangya Hospital of Central South University between May 2017 and October 2021 was conducted. We enrolled all cases with evidence of mediastinal lesions or enlarged mediastinal lymph nodes through a chest computed tomography (CT) scan and/or positron emission tomography/computed tomography (PET/CT).

### Inclusion and exclusion criteria

Inclusion criteria: (1) CT or PET/CT showing mediastinal lesions or enlarged mediastinal lymph nodes; (2) EUS-FNA/FNB examination and cytological smear and cytopathological analysis specimens; (3) The clinical data and follow-up data were complete.

Exclusion criteria: Incomplete clinical data and/or follow-up data.

### Equipment

Olympus EU-ME2 ultrasound endoscope system, ultrasound frequency 6.0 MHz and 7.5 MHz, UCT-260 line scan ultrasound endoscope, 22 Gauge Boston Scientific Fine Needle Aspiration (FNA), 22 Gauge Cook Pro-core Fine Needle Biopsy (FNB).

### Surgery

Preoperative fasting and water deprivation for 8 h; routine testing of coagulation function, routine blood tests and electrocardiogram; excluding patients with severe cardiopulmonary disease and coagulation dysfunction; and aspirin cessation for 1 week before surgery. A signed consent form before surgery was obtained. Intravenous anesthesia and vital signs during surgery were monitored.

### Surgical method

Regular line-array endoscopic ultrasound scans, the decision to perform tissue acquisition technique (FNA or FNB) was at the discretion of the endoscopist. There were many factors for the decision such as the small tumour size (mass size not exceeding 4 cm chose FNB), and some endoscopist preferred biopsy needle but aspiration needle in limited budget patients. Or certain neoplasms, such as lymphoma and stromal tumors, can be assessed by EUS-FNB to confirm the diagnosis. At the same time, when EUS-FNA/FNB is negative or uncertain, elastography and contrast-enhanced ultrasound were used to understand the blood supply and hardness of the lesion to improve the diagnosis rate of selecting the most suspicious target area. Under ultrasound guidance, the puncture needle is pierced into the lesion, avoiding blood vessels and important organs, using slow pull or 5 ml negative pressure (used when micronegative pressure is not sufficient for obtaining tissue), and repeatedly punctured and sampling the lesion 10–13 times before pulling out the puncture needle. The gross tissue specimen was initially evaluated by an experienced endoscopist for adequate tissue sample (white or yellowish tissue at least 2 mm). This procedure is defined as macroscopic onsite evaluation (MOSE). A few tissue smears are used for cytology and DNA ploidy testing. The rest of the tissue strips are fixed in 10% formaldehyde and sent for histopathological examination. Depending on the clinical context, generally 2–4 needles are used per procedure. During the operation, changes in the patient's vital signs, blood oxygen saturation and complexion were closely observed. After the puncture, we observed whether the puncture point showed any bleeding and dealt with it if necessary.

### Postoperative treatment and observation

After the operation, the patient was transferred to the recovery room until fully awakened. The patient was instructed to fast for 6 h and use hemostatic and antibiotics appropriately. We paid attention to whether the patient had chest pain, fever, hematemesis, etc.

### Readouts

(1) EUS-FNA/FNB result judgment: EUS imaging features will be judged for the first time by an experienced endoscopist, and then another expert endoscopist will describe and judge the features of each EUS image again later. When there is disagreement, a final diagnosis will be made after consultation. The inspection results were divided into 4 categories: (1) malignant (see cancer cells); (2) suspected malignant (see suspicious cells); (3) atypical cells; (4) no clear malignant evidence (see inflammatory cells or lymphocytes); categories 1–3 were judged as mediastinal malignant tumors, and category 4 was judged as benign mediastinal lesions.
(2) Follow-up and final diagnosis: A main investigator followed up all patients or their families by telephone. The final diagnosis was made by surgical pathology or follow-up: for those undergoing surgery, the postoperative pathological results were the final diagnosis. If the superficial lymph nodes were completely removed by surgery or malignant cells were found by puncture again, the final diagnosis was malignant. If the follow-up was more than 6 months without disease progression or other treatments, such as anti-tuberculosis, were effective, the final diagnosis was benign lesions.

### Ethics approval and consent to participate

Approval for this study was obtained from the Ethics Committee of The Third Xiangya Hospital of Central South University. All experiments were performed in accordance with relevant guidelines and regulations.

### Statistical methods

SPSS 20.0 software was used for statistical analysis. With the final diagnosis as the gold standard, the sensitivity, specificity, positive predictive value and negative predictive value of the EUS-FNA (FNB) diagnosis were calculated, and Fisher's exact probability method was used for the comparison of rates. *P* < 0.05 was considered statistically significant.

## Results

### General situation

This study included 107 patients (76 men, 31 women, age 56 ± 12 years old). Among all patients, 32 had cough and sputum, 15 had chest pain or chest and back pain, and 10 had partial obstruction and had difficulty swallowing after eating. Shortness of breath occurred in 14 cases, and there were 10 cases of abdominal pain and abdominal distension, 10 cases of fever, 3 cases of fatigue and anorexia, 4 cases of hemoptysis, 1 case of pain under the xiphoid process, 1 case of night sweats, 1 case of periumbilical pain, and 7 asymptomatic cases where a physical examination revealed a lung or mediastinal space-occupying lesion.

### Pathological results of the mediastinal lesions

Among the 107 cases of mediastinal puncture, 35 cases were benign mediastinal lesions and 68 were malignant mediastinal lesions, as shown in Tables 1, 2, respectively.

**Table 1:** Pathological results of benign mediastinal lesions.

Lesions result	Number of cases	Percentage
Tuberculosis	16	49
Sarcoidosis	5	15
Hamartoma	1	3
Stromal tumor	1	3
Other (including abscess, inflammation, fungal disease, etc.)	10	30
Total	33	100

**Table 2:** Pathological results of malignant mediastinal space.

Lesions result	Number of cases	percentage
Lung cancer	54	77.2
Gastric metastatic carcinoma	1	1.4
Esophageal metastatic carcinoma	3	4.3
Mediastinal malignancy	4	5.7
Lymphoma	2	2.9
Thyroid metastatic carcinoma	1	1.4
Prostate-derived metastatic carcinoma	1	1.4
Bile duct metastatic carcinoma	1	1.4
Metastatic cancer of unknown site	3	4.3
Total	70	100

### The diagnostic accuracy of EUS-FNA/FNB for mediastinal space-occupying lesions

EUS-FNA (FNB) examination results: 55 of the 107 patients underwent EUS-FNA examination and 52 underwent EUS-FNB examination. The total sensitivity, specificity, positive predictive value, and negative predictive value of EUS-FNA/FNB for mediastinal space-occupying lesions were 92.00%, 100%, 100%, and 85%, respectively.(as shown in Table 3).

**Table 3:** The diagnostic accuracy of EUS-FNA/FNB for mediastinal space-occupying lesions.

EUS-FNA/FNB diagnosis	Final diagnosis		
	Malignant lesions	Benign lesions	Total
Malignant lesions	68	0	68
Benign lesions	6	33	39
Total	74	33	107

### Examination results of EUS-FNA for mediastinal space occupation

The sensitivity, specificity, positive predictive value, and negative predictive value of EUS-FNA for mediastinal malignant space-occupying lesions were 92.00%, 100%, 100%, and 86.00%, respectively.

### Examination results of EUS-FNB for mediastinal space occupation

The sensitivity, specificity, positive predictive value, and negative predictive value of EUS-FNB for mediastinal malignant space-occupying lesions were 92.00%, 100%, 100%, and 82.00%, respectively.

### Cytological examination, histological examination, combined examination of the two and DNA ploidy detection for the diagnostic efficacy of mediastinal lesions

If cancer cells, suspicious cancer cells, or atypical cells are found in cytology or histology, the lesion was judged to be malignant; no cancer cells, suspicious cancer cells or atypical cells are deemed benign. Abnormal DNA ploidy cells were judged to be malignant. (as shown in Table 4).

**Table 4:** The diagnostic efficacy of DNA ploidy detection, cytological examination, histological examination and cytohistological joint examination for mediastinal lesions.

Diagnosis method	Final diagnosis			
	Sensitivity (%)	Specificity (%)	Positive predictive value (%)	Negative predictive value (5)
Cytology	86 (54/63)	93 (25/27)	100 (54/54)	75 (24/32)
Pathology	89 (56/63)	93 (25/27)	100 (57/57)	78 (25/32)
DNA ploidy detection	71 (45/63)	81 (22/27)	92 (45/49)	60 (24/40)
Cell + pathology combination	90 (57/63)	100 (27/27)	100 (57/57)	81 (26/32)

### The relationship between EUS imaging characteristics and benign or malignant lesions

A total of 107 patients with complete ultrasound gastroscopy reports were analyzed. The EUS imaging features of the malignant mediastinal lesions showed whether the boundary was clear, whether the echo was uniform, and whether the echo was high or low. Compared with benign mediastinal lesions, the difference was statistically significant (*P* < 0.05). (as shown in Table 5).

**Table 5:** The relationship between ultrasound imaging features and benign and malignant lesions.

		Malignant lesions	Benign lesions	*χ* ^2^	*P*
Boundary	clear	13	21	22.343	0
	Not clear	61	12		
Are the edges regular?	Regular	28	15	0.551	0.524
	Irregular	46	18		
Whether to merge?	Fusion	28	14	0.201	0.673
	Not fusion	46	19		
Is the echo uniform?	Yes	18	18	9.337	0.004
	no	56	15		
Echo intensity	Low echo	58	32	5.903	0.020
	Nonhypoechoic	16	1		
Long trail	≤3 cm	17	12	2.071	1.164
	>3 cm	57	21		

### The diagnostic value of endoscopic ultrasonic elastography for mediastinal space-occupying lesions

For the 78 patients with elastography images, the results were scored by a modified 5-scale elastic score (6): the target area and other reference areas are uniformly green is scored as 1; a mosaic pattern of green, yellow, and red is scored as 2; blue and other colors are mixed to form a honeycomb structure is scored as 3; a uniform blue is scored as 4; if there is a large red or green area in a mainly blue area it is scored as 5. Lesions with a score of 1–2 are judged to be benign lesions, and 3–5 are classified as malignant lesions. The diagnostic sensitivity, specificity, positive predictive value, and negative predictive value of elastography for mediastinal space-occupying lesions were calculated to be 95%, 14%, 75%, and 50%, respectively. (as shown in Table 6).

**Table 6:** Diagnostic value of ultrasound gastroscopic elastography for mediastinal space-occupying lesions.

Elastography diagnosis	Final diagnosis		
	Malignant lesions	Benign lesions	Total
Malignant lesions	54	18	72
Benign lesions	3	3	6
Total	57	21	78

### The blood supply characteristics of mediastinal space-occupying lesions with contrast-enhanced ultrasound

Based on the results of contrast-enhanced ultrasound in 50 patients, this study analyzed the blood supply observed on contrast-enhanced ultrasound and summarized its characteristics. The malignant lesions and benign lesions were 90% and 10% in 31 rich blood supply cases. Then, 58% and 42% in 19 absent blood supply cases, respectively. (as shown in Table 7).

**Table 7:** Blood supply and benign or malignant lesions under contrast-enhanced ultrasound.

Contrast Ultrasound	Malignant lesions	Benign lesions	Total
Rich blood supply	28	3	31
Absent blood supply	11	8	19
Total	39	11	50

### Complications

In addition to the discomfort caused by conventional gastroscopy in all patients after EUS-FNA/FNB, 1 patient had dull retrosternal pain, 1 had throat pain and 2 had abdominal pain. No special treatment was administered, and the symptoms disappeared 1–2 days after the operation. One case of mediastinal cyst showed an increase in the infection index after puncture, which improved after anti-infection treatment. No complications, such as damage to surrounding large blood vessels, nerves, or bleeding, were observed.

## Discussion

This study retrospectively analyzed the clinical data of patients with mediastinal space-occupying or mediastinal enlarged lymph nodes in whom EUS-FNA/FNB was performed in our hospital. We found that EUS-FNA/FNB played an important role in the diagnosis of mediastinal space-occupying lesions (including benign and malignant space-occupying lesions). The diagnostic sensitivity and specificity are high, and postoperative complications are few, suggesting that EUS-FNA/FNB has high diagnostic value and safety for mediastinal space-occupying lesions.

It is notable that distinguishing benign and malignant was difficult if only based on cytology. Certain tumors, such as lymphoma and stromal tumors, require histopathological examination to assess changes in their tissue structure and cell morphology to confirm the diagnosis (7). Using tissue needles (ProCore, EUS-FNB) for biopsy could obtain more tissue strips. A multicenter study showed that the sensitivity of EUS-FNB in malignant tumors was 90.2%, the specificity was 99%, the positive predictive value was 100%, the negative predictive value was 78.9%, and the accuracy rate was 92.9% (7). A prospective controlled study of 36 solid pancreatic lesions showed that the diagnostic rates of FNB and FNA were both 83.3%, but FNB required fewer punctures than FNA to obtain satisfactory specimens (8). In this study, EUS-FNA and EUS-FNB had high diagnostic sensitivity, specificity, positive predictive value, and negative predictive value for malignant mediastinal masses, and there was no significant difference between them.

Analysis of the final pathological results of 107 patients found that the 70 malignant lesions were mainly lung cancer or metastatic lung cancer (77.2%, 54/70), among which lung adenocarcinoma was most common. This means that EUS-FNA/FNB is effective in identifying lung cancer (9, 10). The 33 cases of benign lesions were primarily mediastinal tuberculosis (49%, 16/33). Tuberculosis is mainly transmitted through the lymphatic system. Although clinical manifestations, laboratory indicators and imaging findings may indicate tuberculosis, the diagnosis requires pathological examination. Puri et al. (11) suggested that in high-risk areas of tuberculosis, EUS-FNA is an accurate, safe, and minimally invasive way to assess suspected tuberculosis patients with mediastinal lymphadenopathy.

In this study, there were 4 cases of mediastinal malignant masses that were not diagnosed by EUS-FNA/FNB, including 1 case of esophageal tumor and 3 cases of lymphoma. Among them, 3 cases of lymphoma were diagnosed by pathology after complete lymph node resection. The diagnosis of lymphoma require core tissue sample and immunohistochemistry staining, if EUS-FNA/FNB cannot obtain a core tissue sample the diagnosis of lymphoma would be difficult, which is a limitation of EUS-FNA/FNB for the diagnosis of lymphoma.

This study also compared the diagnostic efficacy of cytology, histology and their combined examination for mediastinal lesions and found that the sensitivity, specificity, positive predictive value, and negative predictive value of the two combined examinations were higher, which is consistent with the results of Rong's (12) research. This may be related to the fact that cytology can only provide cytological characteristics but cannot assess the tissue structure. DNA ploidy detection has also been an effective clinical screening method for detecting tumors in recent years. In biological cells, DNA changes regularly with the operation of cell cycle, and always keeps constant content. Except for germ cells, the number of chromosomes in normal human cells is diploid. However, when cells are stimulated by carcinogens or DNA is damaged, cell gene mutation and chromosomal abnormalities will occur, and the number of DNA and chromosomes will change in the process of cell proliferation, that is, single or multiple chromosomes will become aneuploid, which will change the original biological characteristics of cells (13), The change of chromosome number of tumor cells with abnormal DNA content leads to the change of expression of some structural genes (oncogenes), which leads to uncontrolled cell proliferation, resulting in malignant transformation of cells and formation of cancer (14). The number of DNA ploidy can reflect the change of chromosome number, thus playing a role in monitoring the malignant transformation of cells. In this study, after collecting the pathological specimen, cytological thin sections are prepared for Feulgen staining. Detect DNA ploidy through automatic cell DNA ploidy analyzer. No aneuploid cells are benign, and DNA aneuploid cells are malignant. DNA ploidy detection had lower diagnostic value for mediastinal lesions than cytology and histology. Kaur's (15) study showed that the sensitivity, specificity and accuracy of DNA ploidy detection in the diagnosis of cancer are lower than those of cytological examination, but it can be used as an auxiliary to cytological examination to reduce false negatives.

There are different conclusions about the imaging characteristics of malignant mediastinal masses. Studies have found that the prediction rate of imaging features for cancerous lesions is as high as 95%, which can avoid unnecessary biopsy and surgical exploration to a certain extent. Some researchers believe that the ultrasound imaging features of mediastinal malignant tumors are hypoechoic, clear borders, round outlines, uneven echoes, etc (16, 17) and solid and blood-rich masses are more likely to be diagnosed as malignant lesions (18). This study showed that the EUS imaging features of malignant mediastinal masses were unclear boundaries, uneven echoes, and predominantly hypoechoic. Compared with benign masses, the difference was statistically significant.

In recent years, while performing EUS-FNA/FNB clinically, two ultrasonic diagnostic techniques, endoscopic elastography and contrast-enhanced ultrasound, have been added, further improving the diagnosis rate. Ultrasound elastography mainly uses the modified 5-point method6 to judge the hardness of the lesion. When EUS-FNA/FNB is negative or uncertain, the accuracy is improved by selecting the most suspicious target area (19). The results of elastography of 78 patients in this study suggested that the texture of malignant mediastinal space-occupying lesions is relatively hard, which has a high guiding value for diagnosis. Contrast-enhanced ultrasound applies a contrast agent to enhance the backscattered echo. It can display the color and power Doppler signals of the blood vessels, which can significantly improve the resolution and sensitivity of ultrasound diagnosis (20). This study analyzed the results of contrast-enhanced ultrasound in 50 patients and concluded that mediastinal malignant space-occupying lesions usually have a rich blood supply. This may be related to angiogenesis as the basis and prerequisite for tumor growth and metastasis (18, 21).

There are studies showing that negative pressure aspiration cannot improve the quality of EUS-FNA/FNB puncture samples (22). Alizadeh et al. (23). compared slow-pull and negative pressure aspiration (10 ml or 20 ml) to puncture 93 cases of pancreatic space-occupying lesions and found that compared with negative pressure aspiration, the slow-pull method was 90% accurate, and the blood contamination of the sample was light. This study compared the effects of different negative pressure aspirations, whether to apply tissue needles and other factors, on the diagnosis of EUS-FNA/FNB. We found that the use of slow pull could obtain satisfactory tissue specimens, but it was not possible to obtain satisfactory tissue specimens under slight negative pressure. When there were enough tissue strips, 5 ml negative pressure could be used to obtain satisfactory specimens. Among the 107 patients in this study, EUS-FNB (*n* = 52) and EUS-FNA (*n* = 55) showed no significant difference in the diagnostic sensitivity of malignant space-occupying lesions, which is similar to the results of Bang et al. (24).

In summary, EUS-FNA/FNB has high diagnostic sensitivity, specificity, positive predictive value, negative predictive value, and few complications for evaluating mediastinal space-occupying lesions. EUS-FNA and EUS-FNB have high diagnostic sensitivity, specificity, positive predictive value, and negative predictive value for mediastinal space-occupying lesions. There was no significant difference in the comparison between the diagnostic values.

## Data Availability

The original contributions presented in the study are included in the article/Supplementary Material, further inquiries can be directed to the corresponding author/s.
